# Large Language Model–Assisted Review of Clinical Trial Informed Consent for Data Reuse: Retrieval-Augmented Generation Approach

**DOI:** 10.2196/95417

**Published:** 2026-10-15

**Authors:** Lameck Mbangula Amugongo, Lena Schaller, Helene Wendt, R Maarten van Dijk, Enrica Zanuttigh, Claudia Neumann, Andreas Freisinger, Jaroslaw Deska

**Affiliations:** 1 Biostatistics and Data Sciences Department, Boehringer Ingelheim Pharma GmbH & Co. KG, Birkendorfer Str. 65, Biberach, Baden-Wuerttenberg, 88397, Germany, 49 (7351) 54-17807; 2 Staburo GmbH, Munich, Bayern, Germany

**Keywords:** large language model, LLM, GPT-4, retrieval-augmented generation, RAG, informed consent form, ICF, clinical trials, AI, health care

## Abstract

**Background:**

Regulatory frameworks such as the Belmont Report, the Common Rule, and the Declaration of Helsinki require informed consent to ensure participants understand a study’s purpose and can make voluntary decisions about their involvement. Regulations, including the General Data Protection Regulation (Regulation [EU] 2016/679), further emphasize that consent must be freely given and revocable without disadvantage. Although informed consent forms (ICFs) are intended to be clear and accessible, they have become increasingly lengthy and complex. Large language models (LLMs) offer the potential to navigate and interpret this complexity and have shown promise in biomedical information extraction tasks. However, their susceptibility to hallucinations limits their reliability in high-stakes settings. Retrieval-augmented generation (RAG) can mitigate such errors.

**Objective:**

This study evaluates the integration of GPT-4.1 with RAG for reviewing data reuse language in ICFs and its ability to interpret complex ICF language.

**Methods:**

First, we processed 438 ICFs from different trials, including multiple countries, languages, and versions of ICFs. Using expertly curated prompts, we extracted information about data reuse using GPT-4.1. By comparing the LLM-generated data reuse outputs with human expert ground truth, we evaluated accuracy and the time required to extract information for each ICF. To further validate the workflow, we evaluated an independent set of 488 ICFs spanning additional trials, languages, and regions. During the validation phase, the workflow was evaluated using a baseline assessment (89 ICFs) followed by 3 iterative optimization rounds assessed on 14, 281, and 104 ICFs, respectively. For this cohort, we assessed the correctness of LLM outputs along with the quality of supporting evidence provided by the model.

**Results:**

Across the initial set of 438 ICFs, the system achieved 81.6% accuracy. During validation, the baseline accuracy using the preoptimized prompts was 84.0% (89 documents). Accuracy improved sequentially across the 3 iterative optimization rounds, increasing from 82.3% in the first round (14 documents) to 90.7% in the second round (281 documents), and reaching 92.1% in the third and final round on an independent holdout cohort of 104 documents. Ultimately, using our RAG-based approach, the system accurately extracted data reuse information across multiple languages.

**Conclusions:**

Our RAG-based workflow demonstrated the ability to effectively support the ICF review process for data reuse and interpret complex consent language, contributing to improved consistency in ICF evaluations for data reuse.

## Introduction

### Background

Informed consent forms (ICFs) are crucial for studies that involve human participants, safeguarding the well-being, rights, and safety of clinical study participants [1]. ICFs serve as essential documentation that clinical researchers often consult to verify what participants have agreed to regarding the use of their data collected during a study. This includes samples pertaining to biomarkers, imaging, genomic information, and other collected data that can be used for translational science or other scientific improvements.

An ICF contains information on how data will be used within the context of a study (primary use), as well as the purposes for which, and the parties with whom, the data can be shared for further use (data reuse). Reviewing information about data reuse in ICFs is a manual process, involving a dedicated team that assesses ICFs one-by-one to verify data reuse permissions and to distinguish them from the guidelines for primary use. This manual process is tedious and time-consuming, requiring reviewers to navigate complex language and nuanced variations across countries and languages to extract relevant information about data reuse, a challenge that becomes even more pronounced in multinational clinical trials, where multiple country-specific versions of the same ICF further increase review complexity.

Without relevant information from ICFs about data reuse, participants’ data cannot be reused beyond the purpose of the study for which it was collected, hindering advancements in translational medicine and other scientific research. The recent development of large language models (LLMs) presents opportunities to navigate the complex language in informed consent using AI. LLMs have shown significant potential in similar biomedical tasks such as information extraction, authoring clinical documents, and patient trial matching [2]. However, LLMs are prone to hallucination, generating misleading, false, or nonexistent information [3]. To solve this problem, retrieval-augmented generation (RAG) has been proposed [4]. A recent survey has shown that RAG has the potential to reduce hallucinations in LLMs, especially in critical sectors such as health care [5].

The ability of LLMs to navigate the intricate and specialized language within ICFs remains largely unexplored. Shi et al [6] have shown that LLMs can generate ICF documents with improved readability, understandability, and actionability without sacrificing accuracy or completeness. Similarly, InformGen was proposed as a specialized LLM pipeline for ICF generation, achieving near-perfect compliance, high accuracy (80%‐95%), and editable evidence within a human-in-the-loop framework [7]. In another study, a chat-based application was created to enable users to interact with ICF documents, thereby providing precise and prompt answers about data reusability [8]. The proposed chatbot is limited to only one ICF document at a time and requires users to type their questions. This can lead to inconsistencies in LLM responses, as each user may write their prompts differently.

In this study, we investigated the integration of LLMs into the clinical workflow for ICF review, focusing on their capacity to comprehend and process the complex linguistic structures inherent to these documents. By leveraging a RAG-like pipeline and expert-defined prompts, our findings demonstrate that LLMs are capable of extracting relevant information related to data reuse. Our RAG-based application can process one or multiple ICF documents at a time, improving the efficiency of ICF document analysis.

### State of the Art

A recent systematic review highlights an increasing number of studies exploring the use of RAG-based LLMs across diverse aspects of clinical workflows [5]. However, the use of RAG-based LLMs in the informed consent process is relatively new. To date, only a few studies have explored the potential for LLMs to process ICF documents. Most of these studies focus on LLM-generated ICF content, analyzing readability, accuracy, and ethical implications. For example, early research by Decker et al [9] demonstrated that LLM-based chatbots can outperform traditional surgeon-authored consent materials in terms of clarity and completeness, particularly when describing surgical benefits and alternatives. These findings suggest that LLMs, when appropriately integrated into the consent workflow, may enhance participant understanding and reduce the risk of misinformation. In a recent study, Vaira et al [10] assessed the quality and readability of ICFs generated by various chat models, such as GPT-4 [11] and Bard Gemini [12]. Their results show that GPT-4 generated the highest-rated consent documents, demonstrating AI’s promise in improving patient communication. In another study, Shi et al [6] reported that LLM-generated ICFs are comparable to human-authored documents in terms of accuracy and completeness, with significantly improved readability and actionability.

Raimann et al [13] evaluated the performance of 3 chat models, such as GPT-3.5, GPT-4, and Gemini, in creating information sheets for 6 common anesthesiologic procedures, guided by patients’ questions. Multiple drafts of anesthesia forms were created and refined, with results evaluated against established standard checklists. While the study illustrated the potential for the application of AI in informed consent sheets, it also underscored a critical limitation: none of the tested models were able to produce legally compliant informed consent sheets without human oversight. This highlights the current boundary of LLM capabilities and the necessity of hybrid approaches that combine machine efficiency with human judgment.

Beyond technical performance, the emerging literature has begun to address the relational and ethical dimensions of LLM use in informed consent. Rudra et al [14] outlined the challenges associated with surgical informed consent, highlighting both the potential benefits and limitations of LLMs, as well as the ethical implications of simulated empathy. They found that LLMs may help detect signs of patient distress and alert health care providers, thereby supporting more timely and empathetic communication. These perspectives reinforce the notion that LLMs should augment, not replace, human interactions, preserving the trust and emotional nuance essential to ethical consent processes. Finally, Allen et al [15] introduced a conceptual framework for informed autonomy, arguing that, while LLMs can enhance information processing and values clarification, they also risk undermining patient agency if not carefully deployed. Their proposed guidelines for implementation reflect a growing consensus that ethical integration of LLMs in health care requires ongoing evaluation, transparency, and patient-centered design [5].

## Methods

### Study Design

The advent of LLMs has transformed natural language processing, shifting natural language processing from task-specific representations to task-agnostic pretraining [16]. LLMs have demonstrated performance comparable to humans on many different tasks [17,18]. However, LLMs are static snapshots with knowledge cutoff dates; for example, Google Gemini 2.5 Pro incorporates data up to January 2025 [19], and the cutoff for GPT-4.1 is June 2024 [20]. Moreover, LLMs lack access to proprietary information, which includes ICF documents. To address these limitations, we proposed a RAG workflow to augment an LLM with ICF documents containing information about data reuse. The information from the ICF documents minimizes hallucinations, enabling the LLM to generate reliable responses supported by extracted text that acts as a verifiable reference for the information the LLM used to generate the outcome.

### RAG Workflow

Figure 1 shows the RAG workflow. The process begins with the user uploading an ICF document (or multiple documents). The uploaded ICF is then ingested into an in-memory vector store. Afterward, we perform a context-based hybrid search (keyword and semantic) to retrieve relevant information from the ICF documents. The relevant content is then passed to the LLM with expert-defined prompts. Finally, the LLM-generated outcome for data reuse is presented to the user as a response to aid decision-making.

**Figure 1.**
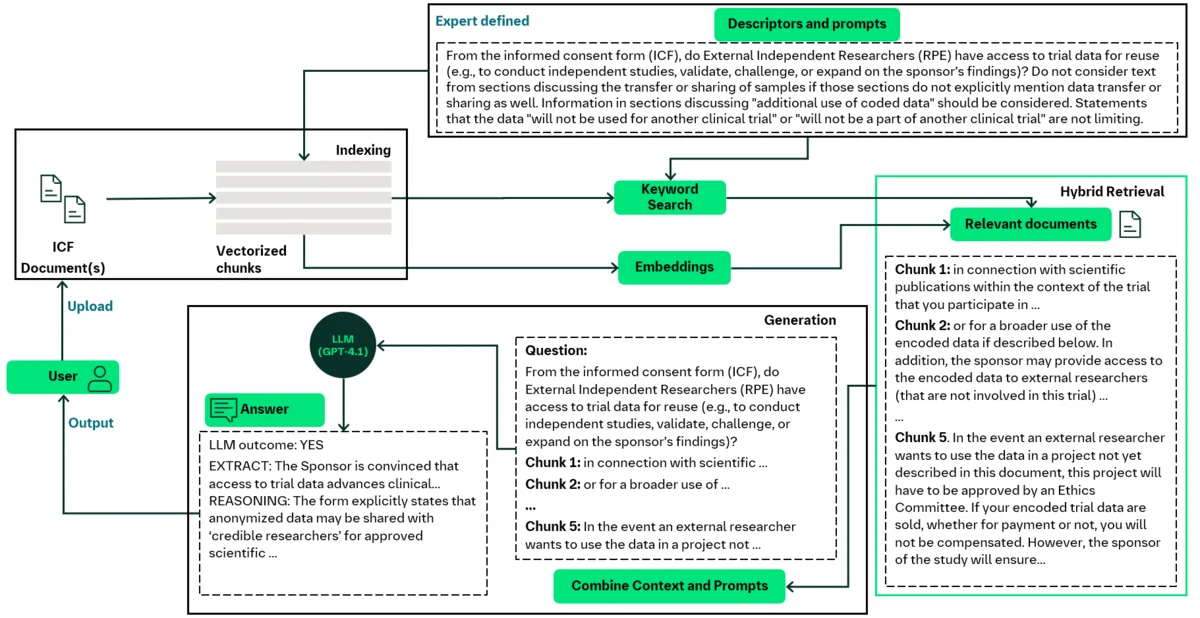
Workflow illustrating the schematic retrieval-augmented generation (RAG) pipeline. Uploaded informed consent form (ICF) documents are chunked and indexed, and then queried using hybrid retrieval (keyword+embeddings) guided by expert-defined prompts. Retrieved chunks are combined with the query and passed to the large language model (LLM), which generates a structured answer with evidence and reasoning returned to the user.

### Vector Database

To securely and efficiently process uploaded ICF documents, we implemented an in-memory vector store. First, text was extracted from the ICF document and segmented through a process called “chunking.” Chunking is necessary because a single ICF document typically contains at least 15 pages, and embedding models are limited by the number of tokens they can process within a single sequence. To determine the optimal chunking strategy, we tested different chunk sizes of 500, 1000, and 2000 tokens. After chunking, the text segments were transformed into high-dimensional embeddings using the OpenAI text-embedding-3-small model [21] to capture the semantic meaning of the ICF document. The text-embedding-3-small model was chosen because of its high efficiency and better performance than predecessor models, such as text-embedding-ada-002 [21].

The text embeddings, together with associated metadata, were then instantiated within the LangChain MemoryVectorStore, allowing for rapid similarity search and contextual retrieval of relevant consent clauses. The in-memory architecture was deliberately chosen to ensure compliance with data minimization principles, averting persistent storage of ICF information, thereby aligning with ethical standards in research data governance and regulations [22], while also maintaining technical efficiency in semantic retrieval. Figure 2 shows an example representing 3 chunk embeddings.

**Figure 2.**
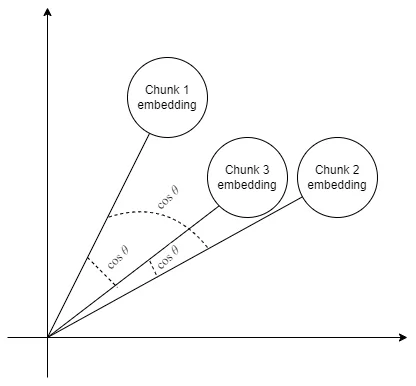
An example of embedding-based representation of 3 chunks, where each chunk is mapped to a vector in semantic space. Cosine similarity scores quantify the relevance between a query and each chunk embedding, enabling identification and ranking of the most similar chunks based on semantic proximity.

### Prompting Strategy

Prompting is fundamental to guide LLMs in generating outputs. In a nutshell, a prompt is a set of instructions that help the model comprehend the type of response expected by the user. There are different prompting strategies aimed at improving prompt clarity, contextual alignment, and structural flow to provide the most accurate response to the user’s query [23]. In this study, we explored different prompting strategies, that is, zero-shot and few-shot prompting, with zero-shot prompting, combined with clear rules, chosen as the final strategy because it is fast and requires fewer tokens. This helps the model comprehend which text to look for in the ICF document and the format in which the model should provide the responses. An example of zero-shot prompting is provided in Textbox 1.

The full list of prompts is provided in Multimedia Appendix 1 (Table S1). The descriptors COUNTRY, TRIAL_NO, VERSION, and VERSION_DATE were not assessed, as they do not affect data sharing.

Textbox 1. **An example of a data-sharing prompt (MED_TR_REL) to extract relevant information on whether the patient has consented to allow their data to be used to improve the trial medication and related medications, with defined qualifications for outputs of YES, NO, and N/A (not applicable).**

**Example**

From the information provided in the informed consent form (ICF). Can trial data be used to improve medication quality and related substances? This info is in the confidentiality and data privacy section. Look for phrases such as “understand how the trial drug and similar drugs work in the body and the study drug mode of action.”

If it is mentioned that data can be reused to understand how a similar trial drug works or better understand related diseases, then reply YES.

If it is explicitly mentioned that data “cannot” be used to improve related medication or similar trial drugs, reply NO.

If it is not explicitly mentioned that data can be used to understand similar trial drugs or related medication, or no information is available, reply N/A. Extract information from the document to support the answer. Descriptor: MED_TR_REL

<excerpt from the informed consent form>

{excerpt}

</excerpt from the informed consent form>

### Retrieval

To ensure accurate and contextually relevant extraction from ICFs, we used both preretrieval and postretrieval techniques. Preretrieval optimization focused on indexing strategies, including segmentation of the uploaded ICF into granular text chunks, alignment of text with associated metadata, and embedding of prompts into vector space representations. For retrieval, we used a hybrid approach, combining similarity search and keyword search. First, we performed a similarity search on the vector store, returning the top-*k* (*k*=5) most relevant segments. Next, a BM25 (best matching 25) retriever was applied to the same indexed text and metadata to perform a keyword-based search, likewise retrieving the top 5 results. The final number of unique chunks passed to the LLM context window depends on the degree of overlap between the 2 retrieval methods. Postretrieval, the outputs of both methods were integrated into an ensemble retriever, which combined the BM25 keyword search and vector similarity search with weighted contributions (0.3 and 0.7, respectively). This hybrid retrieval strategy allowed us to balance lexical precision with semantic nuance in the ICF language, thereby improving the quality of the retrieved information and ensuring that both explicit terminology and contextual meaning within the ICF were captured. Lastly, no explicit translation pipeline was applied; multilingual semantic retrieval was achieved directly through the embedding model. The retrieved chunks were provided in the original language, and the LLM was instructed to provide its reasoning in English.

### LLM

Initially, we explored several models, such as GPT-3.5 and GPT-4o. However, after the release of GPT-4.1, which introduced major improvements such as a long context window [20] and instruction following, we opted to use GPT-4.1 in our RAG pipeline. To improve LLM generation, we developed a structured workflow using LangChain to create a RAG pipeline that integrates information retrieval with LLM outcome generation for data reuse. For each prompt, the system first retrieved the most relevant sections from the ICF, which served as the external knowledge source from which the LLM derived its data reuse assessment. Model inference parameters were configured with a low temperature (0.1) to ensure deterministic and consistent outputs, with caching disabled to avoid reuse of prior responses across runs, and with a high maximum token limit (16,000) to fully leverage the model’s long-context capabilities for processing lengthy ICF documents. For structured and reliable outputs, we defined a structured data model using Pydantic’s BaseModel class, which provides automatic validation and serialization of fields through type annotations [24]. The extracted data schema specifies 4 components: descriptor, outcome, supporting extract, and LLM reasoning, thereby enforcing consistency in outputs and reducing ambiguity in downstream analysis. Such schema-driven approaches are critical in biomedical research, where methodological rigor and reproducibility are important.

### Application

To enable users to access and apply the workflow without the need to write or execute code, we developed a web-based application using Streamlit [25]. Streamlit was selected due to its simplicity and rapid development capabilities, which allow the efficient creation and deployment of interactive data applications. The final application integrates the RAG-based workflow for analyzing ICFs directly within the Streamlit interface, providing an intuitive and user-friendly environment for performing all analyses. The application supports the processing of one or multiple ICFs; however, each document is processed independently to ensure data integrity. For processing multiple ICFs, a new in-memory vector store is instantiated for each ICF, retrieval is restricted to that document, results are saved immediately upon completion, and the vector store is cleared before processing the next document, thereby preventing cross-document context contamination.

### Deployment

Due to the confidential nature of ICFs, data security is essential. In our study, all LLM calls were performed through API calls in Boehringer Ingelheim’s secure environment deployed on Amazon Web Services (AWS) private cloud, upholding data integrity and privacy. The application was deployed on Posit Connect, a platform that enables users to securely share insights, automate tasks, and deploy various data science applications [26]. Within Boehringer Ingelheim, Posit Connect is deployed on a private AWS cloud, allowing users to securely access the system and analyze ICFs.

### Evaluation

To evaluate the performance of our RAG pipeline, we conducted an experiment using 438 ICF documents and tested different chunk sizes of 500, 1000, and 2000 tokens. For the purposes of this project, “ICF document” refers to unsigned ICFs, including the standard informed consent template of a study and associated country-specific forms. Model performance was formally evaluated using accuracy, defined as the correctness of the LLM outcome. Completeness (correctness of the outcome with appropriate supporting evidence) and reasoning quality were assessed qualitatively by expert reviewers and were not quantified using formal metrics. To compute accuracy, we compared the LLM outcome with the ground truth provided by subject-matter experts. Because we did not have ground truth for the supporting text in the ICF documents, human experts manually evaluated the LLM outcome and reasoning. Evaluation was performed only on data-sharing descriptors (Table 1). In total, 21 data-sharing descriptors were analyzed, identifying mentions of intended recipients (ie, LIC, RPC, RPE, SPAFI), reuse purposes (MED_TR, MED_TR_REL, MED_ALL, DIS_ALL, DIS_TR, DIS_TR_REL, DIS_TR_TA, DPROD_ALL, DPROD_TR_REL, TRPROD_ALL, TRPROD_TR_REL, QUAL_ALL), data retention (DLINK30, RR50, RR80), and content (RESTRICTIVE_WORDING, GENOMIC_INFO).

**Table 1.** Data-sharing descriptors investigated and their definitions^a^.

Descriptor	Definition	Outcome
Recipient		
LIC	Data can be shared with owners of a licensed product	YES|NO|N/A
RPC	Data can be shared with research partners and collaborators of the sponsor	YES|NO|N/A
RPE	Data can be shared with external independent researchers	YES|NO|N/A
SPAFI	Data can be shared with sponsors, affiliates, or third-party processors	YES|NO|N/A
Purpose		
MED_TR	Data can be shared to learn about the trial medication	YES|NO|N/A
MED_TR_REL	Data can be shared to learn about medications related to the trial medication	YES|NO|N/A
MED_ALL	Data can be shared to learn about any medications	YES|NO|N/A
DIS_ALL	Data can be shared to learn about any disease	YES|NO|N/A
DIS_TR	Data can be shared to learn about the disease studied in the trial	YES|NO|N/A
DIS_TR_REL	Data can be shared to learn about diseases related to the trial disease	YES|NO|N/A
DIS_TR_TA	Data can be shared to learn about diseases in a specific therapeutic area	YES|NO|N/A
DPROD_ALL	Data can be shared to develop any diagnostic tools	YES|NO|N/A
DPROD_TR_REL	Data can be shared to develop diagnostic tools related to the trial disease	YES|NO|N/A
TRPROD_ALL	Data can be shared to develop any therapeutics	YES|NO|N/A
TRPROD_TR_REL	Data can be shared to develop therapeutics related to the trial disease	YES|NO|N/A
QUAL_ALL	Data can be shared to improve the quality of future clinical trials	YES|NO|N/A
Data retention		
DLINK30	The subject re-identification link will be deleted after 30 years	YES|NO
RR50	Data will be retained for up to 50 years	YES|NO
RR80	Data will be retained for up to 80 years	YES|NO
Specific text		
RESTRICTIVE_WORDING	Any text with other restrictions on data reuse	YES|NO
GENOMIC_INFO	Any text mentioning genomic data	YES|NO

a Descriptors in the recipient category specify with whom the data can be shared; those in the purpose category define the intended reuse of the data. Descriptors under data retention describe data storage duration and practices, and specific text descriptors capture additional constraints or content (eg, restrictive wording or genomic information). Outcomes indicate whether each descriptor was explicitly supported in the ICF (YES, NO, or not applicable [N/A]).

### Validation and Prompt Optimization

To validate the performance of our RAG pipeline, experts selected 488 ICF documents from different trials, languages, and regions. These ICF documents were unique from those used in the evaluation process. Subject-matter experts established ground truth values and concurrently compared them to the LLM outputs. This approach was necessary for the concomitant prompt optimization process, as it facilitated the identification of recurring misclassifications. Prompts were optimized 3 times over the course of validation to address recurring failure modes, including misinterpretation of exclusion criteria, sensitivity to negation, and inconsistent handling of “not applicable” cases (Textbox 2). The baseline assessment and subsequent iterative optimization steps were performed during routine work by the subject-matter experts on datasets comprising 89, 14, 281, and 104 ICFs, respectively. The first optimization was the addition of text meant to guide the LLM to specific sections of the ICF document with relevant information. The LLM was advised to ignore sections discussing the sharing of biological samples and to prioritize sections that mentioned “coded data.” The following round of optimization featured the creation of a new descriptor designed to identify any version of the blanket statement specifying that data can only be reused: “strictly for the purposes described.” Based on subject-matter expert assessment, such a statement would mean that data reuse is only permissible if it is explicitly mentioned in the ICF. This additional descriptor was not classified as a “data-sharing” descriptor and was used to format a higher-level business rule stating that, if such a sentence is detected, all “N/A” outputs must be converted to “NO” outputs. The final optimization step was the addition of text-specific examples to better define prohibitive and permissive language. The LLM’s responses were assessed qualitatively for completeness and relevance, and quantitatively for accuracy at each optimization stage. Finally, we computed the number of ICFs correctly analyzed for data-sharing descriptors across all 488 validation ICFs, categorized by country of origin.

Textbox 2. **Illustration of the prompt optimization process for the sample descriptor research partner or noncommercial collaborator (RPC). The original prompt text of the RPC descriptor was modified twice (optimization rounds 1 and 3) to include text specifying which informed consent form (ICF) sections to prioritize as well as text-based examples clarifying permissive and prohibitive language. The second round of optimization introduced a new descriptor, STRICTLY, which was used to create an overarching business rule to convert all “N/A” outputs to “NO” outputs if specific phrasing was detected. The text added to the RPC prompt at each optimization step is indicated in bold.**

Original prompt text: **RPC**

From the ICF, does RPC have access to trial data for reuse?

Reply “YES” if access is explicitly granted to research partners or collaborators (eg, “research partners,” “collaborating institutions,” “noncommercial collaborators”).

Reply “NO” only if data access by RPC (eg, “research partners,” “collaborating institutions,” “noncommercial collaborators”) is explicitly prohibited.

Reply “N/A” if access is not mentioned or is unclear or no information is available.

First round of optimization: **RPC**

From the ICF, do RPC have access to trial data for reuse?

**Do not consider text from sections discussing the transfer or sharing of samples if those sections do not explicitly mention data transfer or sharing as well. Information in sections discussing “additional use of coded data” should be considered**.

Reply “YES” if access is explicitly granted to RPC (eg, “research partners,” “collaborating institutions,” “noncommercial collaborators”).

Reply “NO” only if data access by RPC (eg, “research partners,” “collaborating institutions,” “noncommercial collaborators”) is explicitly prohibited.

Reply “N/A” if access is not mentioned or is unclear or no information is available.

Second round of optimization: addition of **STRICTLY** descriptor

From the information provided in the ICF. Does the ICF mention if data can only be used for described purposes?

Answer can be only YES or NO.

If there is a clause for data sharing or use for future research such as the following examples “strictly for the purposes described” or “solely for the purposes described” or “limited to the context of the present study” or “only to answer scientific questions about the study” or “only for the purposes described therein” reply with YES. Else respond with NO.

Third round of optimization: **RPC**

From the ICF, do RPC have access to trial data for reuse?

Do not consider text from sections discussing the transfer or sharing of samples if those sections do not explicitly mention data transfer or sharing as well. Information in sections discussing “additional use of coded data” should be considered.

**Statements that the data “will not be used for another clinical trial” or “will not be a part of another clinical trial” are not limiting**.

Reply “YES” if access is explicitly granted to **partners**, RPC (eg, “**scientific partners**,” “research partners,” “collaborating institutions,” “noncommercial collaborators”).

**The following is an example of text qualifying for a YES reply:**

**“Service providers (such as clients, research partners of clients, and affiliates of client groups) may access your coded data.”**

Reply “NO” only if data access by RPC (eg, “research partners,” “collaborating institutions,” “noncommercial collaborators”) is explicitly prohibited.

Reply “N/A” if access is not mentioned or is unclear or no information is available.

### Data Analysis

For data-sharing information, we were not only interested in the accuracy, completeness, and usefulness of the answers provided by the RAG-based pipeline but also in how the output impacts decision-making regarding data reuse. As such, we computed class-specific and overall accuracies for each data-sharing descriptor extracted from the 488 validation ICFs. ICFs were analyzed in their original languages without explicit translation, relying on the LLM’s multilingual capabilities; this approach reflects common practice and is aligned with the manual review process, in which subject-matter experts either reviewed ICFs in their original language or translated them into English prior to evaluation.

Each descriptor was evaluated across possible output categories, as shown in Table 1. An output of YES indicates that the ICF contains text supporting data reuse for the specified descriptor, while an output of NO reflects that the ICF explicitly prohibits data reuse for that purpose. If no context is provided in the ICF to support or prohibit data reuse, the output for the descriptor would be N/A. However, descriptors identifying clauses for data retention practices (DLINK30, RR50, and RR80), as well as the descriptors pinpointing specific language (GENOMIC_INFO and RESTRICTIVE_WORDING), were evaluated on a binary scale (YES/NO), where an output of NO applies both in the context of an explicit prohibition and in the absence of any mention related to the descriptor. Reporting class-level assessment allowed us to thoroughly examine how well our workflow distinguished affirmative, ambiguous, or negative data-sharing statements, while overall accuracy provided a high-level summary of system performance.

### Ethical Considerations

This study is classified as nonhuman participant research. The evaluators involved were exclusively subject-matter experts who are also listed as co-authors of this work. The reviewers were subject-matter experts with several years of experience performing ICF reviews for data reuse as part of their routine work. Consistency across reviewers was supported using guidelines and structured evaluation criteria. Moreover, no identifiable private information was collected, analyzed, or shared, and no external participants were involved. Thus, this study does not require institutional review board approval.

## Results

### Application Performance and Accuracy

The performance of GPT-4o and GPT-4.1 was comparable (Multimedia Appendix 2). GPT-4.1 outperformed GPT-4o on several descriptors, especially descriptors requiring reasoning and instruction following, such as those identifying further restrictive wording and genomic information. From this point onward, all results were obtained using GPT-4.1 as the LLM. When testing the effect of different chunk sizes (Multimedia Appendix 3), average accuracies of 79.6%, 80.5%, and 81.6% were achieved across all data-sharing descriptors for chunk sizes of 500, 1000, and 2000 tokens, respectively.

To maximize accuracy, a chunk size of 2000 tokens was chosen for subsequent analyses. As shown in the screenshot in Figure 3, the application presents all relevant information to help the user efficiently make a decision about data sharing. Emphasizing user autonomy, the application also allows users to make corrections when the LLM output is wrong. In terms of speed, our application processed ICF documents with an average time of 1.95±0.35 minutes compared to 7.06±3.42 minutes with the manual review process. The full LLM results for an example ICF are provided in Multimedia Appendix 4.

**Figure 3.**
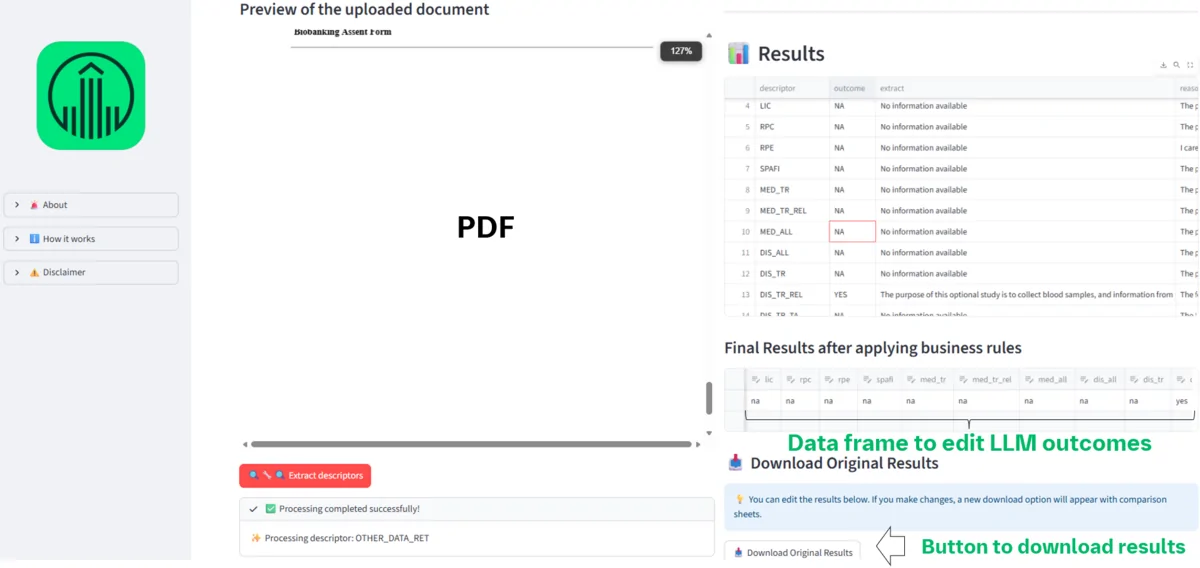
Streamlit application that enables users to access our automatic workflow to analyze informed consent forms (ICFs). The application is intuitive, enabling the user to view the results and uploaded PDF side-by-side. The results can be edited to enable corrections. Once a user is satisfied with the results, the results can be downloaded.

In analyzing 438 ICFs, the system achieved an overall accuracy of 81.6% when compared with subject-matter expert evaluations. However, some shortcomings were identified, such as an accuracy of less than 70% for several descriptors: MED_ALL, DIS_TR_TA, RESTRICTIVE_WORDING, and GENOMIC_INFO. To address these shortcomings, we performed prompt optimization and validated the workflow.

### Validation and Prompt Optimization

For the validation of our workflow, we conducted further analysis of 488 ICF documents through 4 validation rounds. Baseline validation on 89 ICFs achieved an overall accuracy of 84.0%, with notable variability across descriptors, including lower performance for complex descriptors such as RPE (n=66, 74.2%) and RPC (n=71, 79.8%). Iterative prompt refinement resulted in substantial and consistent performance improvements across most descriptors. Following the first prompt update, evaluated on 14 ICFs, performance improved for several descriptors, with accuracies reaching 92.9% (n=13) for RPE and 100% (n=14) for SPAFI and QUAL_ALL, indicating early gains from prompt refinement despite the small sample size. Following the second round of optimization, a second, larger validation on 281 ICFs demonstrated more consistent and generalizable improvements, with many descriptors exceeding 90% accuracy, including RPC (n=263, 93.6%), RPE (n=260, 92.5%), and MED_TR (n=271, 96.4%), although some variability persisted for more ambiguous descriptors. After the final optimization, evaluation on 104 ICFs yielded an overall accuracy of 92.1%, with strong and stable performance across most descriptors, including RPC (n=201, 97.1%), RPE (n=98, 94.2%), and SPAFI (n=102, 98.1%). Improvements were also evident in class-specific accuracies, particularly for YES and N/A predictions, indicating better alignment between prompt instructions and decision boundaries (Multimedia Appendix 5). While a small number of descriptors (eg, MED_ALL, RESTRICTIVE_WORDING) remained challenging, the optimization process enhanced the robustness, consistency, and interpretability of outputs, demonstrating the effectiveness of systematic prompt engineering in improving RAG-based classification performance. The performance improvements for all rounds of optimization can be observed in Table 2.

**Table 2.** Accuracy (ACC, %) of model performance across all data-sharing descriptors over 4 validation stages (baseline and 3 iterative rounds of optimization)^a^.

Descriptor	Baseline ACC (n=89), n (%)	First round ACC (n=14), n (%)	Second round ACC (n=281), n (%)	Third round ACC (n=104), n (%)
LIC	79 (88.8)	12 (85.7)	260 (92.5)	102 (98.1)
RPC	71 (79.8)	11 (78.6)	263 (93.6)	101 (97.1)
RPE	66 (74.2)	13 (92.9)	260 (92.5)	98 (94.2)
SPAFI	79 (88.8)	14 (100)	242 (86.1)	102 (98.1)
MED_TR	78 (87.6)	11 (78.6)	271 (96.4)	98 (94.2)
MED_TR_REL	75 (84.3)	9 (64.3)	228 (81.1)	90 (86.5)
MED_ALL	71 (79.8)	7 (50)	224 (79.7)	62 (59.6)
DIS_ALL	70 (78.7)	10 (71.4)	260 (92.5)	94 (90.4)
DIS_TR	75 (84.3)	13 (92.9)	265 (94.3)	98 (94.2)
DIS_TR_REL	75 (84.3)	11 (78.6)	249 (88.6)	99 (95.2)
DIS_TR_TA	77 (86.5)	10 (71.4)	253 (90.0)	93 (89.4)
DPROD_ALL	76 (85.4)	12 (85.7)	263 (93.6)	98 (94.2)
DPROD_TR_REL	79 (88.8)	13 (92.9)	266 (94.7)	101 (97.1)
TRPROD_ALL	69 (77.5)	12 (85.7)	234 (83.3)	98 (94.2)
TRPROD_TR_REL	83 (93.3)	8 (57.1)	244 (86.8)	93 (89.4)
QUAL_ALL	78 (87.6)	14 (100)	270 (96.1)	102 (98.1)
DLINK30	75 (84.3)	13 (92.9)	270 (96.1)	101 (97.1)
RR50	83 (93.3)	14 (100)	272 (96.8)	90 (86.5)
RR80	79 (88.8)	14 (100)	274 (97.5)	102 (98.1)
RESTRICTIVE_WORDING	43 (48.3)	7 (50)	210 (74.7)	87 (83.7)
GENOMIC_INFO	88 (98.9)	14 (100)	275 (97.9)	102 (98.1)
Average accuracy	84.0	82.3	90.7	92.1

a Overall, average accuracy improved from 84.0% at baseline to 92.1% by the third round, with most descriptors showing substantial gains after iterative refinement.

Overall, validation achieved an average performance of 89.6% in terms of accuracy across all data-sharing descriptors (Table 3). High accuracy (>80%) was observed across most descriptors, with only 2 descriptors, MED_ALL and RESTRICTIVE_WORDING, attaining lower accuracies of 74.6% (364/488) and 71.1% (347/488), respectively.

**Table 3.** The overall performance of the system for all data-sharing descriptors on the full validation corpus of 488 informed consent form (ICF) documents. The accuracy (ACC, as both percentage and fraction) for each output (YES, N/A, or NO), as well as accuracy per descriptor, is presented.

Descriptor	ACC: YES, n/N (%)	ACC: N/A, n/N (%)	ACC: NO, n/N (%)	ACC, n/N (%)
LIC	260/275 (94.5)	189/209 (90.4)	4/4 (100)	453/488 (92.8)
RPC	351/382 (91.9)	93/104 (89.4)	2/2 (100)	446/488 (91.4)
RPE	270/314 (86.0)	147/149 (98.7)	20/25 (80)	437/488 (89.5)
SPAFI	409/418 (97.8)	25/67 (37.3)	3/3 (100)	437/488 (89.5)
MED_TR	384/398 (96.5)	70/86 (81.4)	4/4 (100)	458/488 (93.9)
MED_TR_REL	295/307 (96.1)	103/177 (58.2)	4/4 (100)	402/488 (82.4)
MED_ALL	272/282 (96.5)	88/192 (45.8)	4/14 (28.6)	364/488 (74.6)
DIS_ALL	197/221 (89.1)	210/231 (90.9)	27/36 (75.0)	434/488 (88.9)
DIS_TR	365/379 (96.3)	79/102 (77.5)	7/7 (100)	451/488 (92.4)
DIS_TR_REL	335/341 (98.2)	92/139 (66.2)	7/8 (87.5)	434/488 (88.9)
DIS_TR_TA	321/333 (96.4)	108/151 (71.5)	4/4 (100)	433/488 (88.7)
DPROD_ALL	171/193 (88.6)	265/281 (94.3)	13/14 (92.9)	449/488 (92.0)
DPROD_TR_REL	202/218 (92.7)	253/265 (95.5)	4/5 (80.0)	459/488 (94.1)
TRPROD_ALL	218/242 (90.1)	189/239 (79.1)	6/7 (85.7)	413/488 (84.6)
TRPROD_TR_REL	249/269 (92.6)	175/214 (81.8)	4/5 (80.0)	428/488 (87.7)
QUAL_ALL	265/277 (95.7)	144/148 (97.3)	55/63 (87.3)	464/488 (95.1)
DLINK30	114/129 (88.4)	N/A^a^	345/359 (96.1)	459/488 (94.1)
RR50	7/7 (100)	N/A	454/481 (94.4)	461/488 (94.5)
RR80	77/83 (92.8)	N/A	392/405 (96.8)	469/488 (96.1)
RESTRICTIVE_WORDING	10/108 (9.3)	N/A	337/380 (88.7)	347/488 (71.1)
GENOMIC_INFO	387/395 (98.0)	N/A	92/93 (98.9)	479/488 (98.2)
Overall	89.9	78.5	89.1	89.6

a N/A: not applicable.

In total, of the 488 validation ICFs analyzed, 186 had completely correct outcomes for all data-sharing descriptors. As shown in Multimedia Appendix 6, 441 ICFs from 26 different countries and 47 general template (no country affiliation) ICFs were analyzed. The application produced fully correct outputs for a high proportion of ICFs from certain countries, including Poland (32/39, 82.1% are fully correct) and Norway (10/10, 100% are fully correct). In contrast, fully correct outcomes were much less common for ICFs from the Republic of Korea (6/51, 11.8% are fully correct) and Japan (1/15, 6.7% are fully correct).

### Data Analysis

Across a validation set of 488 informed consent documents, our workflow demonstrated strong performance for most data-sharing descriptors. As shown in Table 2, accuracy improved with each round of prompt optimization. Subsequently, incorrect LLM outputs were reduced over time with iterative prompt optimization, as shown in Multimedia Appendix 5.

Overall, the highest accuracy was achieved for GENOMIC_INFO, which reached 98.2% overall and showed near-perfect detection of both affirmative (387/395, 98.0%) and negative (92/93, 98.9%) cases. QUAL_ALL also performed robustly, with high and well-balanced accuracies across all classes and an overall accuracy of 95.1% (464/488). Several descriptors related to medicinal and disease-specific data reuse, including MED_TR (458/488, 93.9%) and DIS_TR (451/488, 92.4%), exhibited similarly strong performance, supported by perfect accuracy in the identification of NO cases. Descriptors involving broader or more conditional statements showed greater variability. MED_TR_REL (402/488, 82.4%) and DIS_TR_TA (433/488, 88.7%) maintained high accuracy for YES classifications but demonstrated reduced performance for N/A or NO outcomes, suggesting difficulty in interpreting nuanced or context-dependent phrasing. MED_ALL showed the greatest imbalance, with high accuracy for YES cases (272/282, 96.5%) but very low accuracy for NO cases (4/14, 28.6%), resulting in a lower overall accuracy of 74.6% (364/488). The lowest performance was observed for RESTRICTIVE_WORDING, which achieved an overall accuracy of 71.1% (347/488). This descriptor had particularly low accuracy in identifying YES cases (10/108, 9.3%), indicating that restrictive or implicitly limiting statements remain challenging for automated extraction methods.

As observed in Figure 4, the RESTRICTIVE_WORDING descriptor had the largest number of incorrect outcomes, followed by MED_ALL and MED_TR_REL. This suggests that, for these descriptors, subject-matter experts should take additional care when reviewing LLM outcomes. The majority of incorrect outcomes (48.9%) were determined by subject-matter experts to have a ground truth value of “N/A,” which applies in cases where the document does not explicitly mention the conditions for data reuse for the descriptor in question.

**Figure 4.**
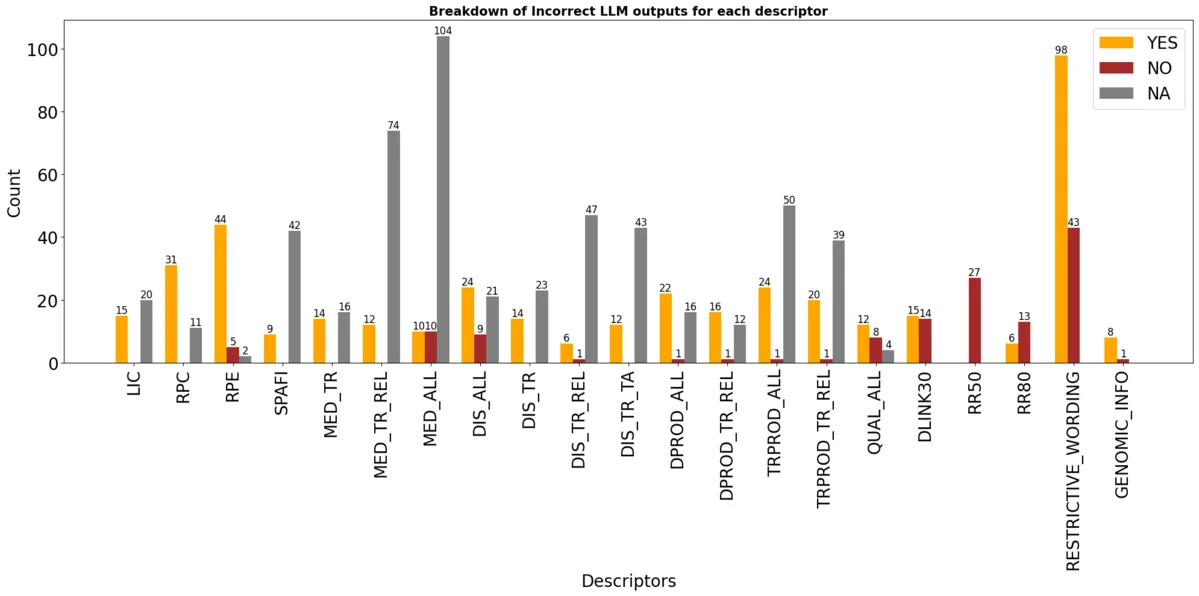
Distribution of incorrect large language model (LLM) outputs for the full validation corpus of 488 informed consent forms (ICFs). For each data-sharing descriptor, the total number of cases where ground truths of “YES,” “NO,” and “N/A” (not applicable) were misclassified is presented.

Overall, the results underscore that LLMs have substantial potential to assist in navigating the complex linguistic structures of ICFs, with several descriptors demonstrating high accuracy and balanced precision-recall performance. These strong outcomes indicate that LLMs with RAG can reliably extract and interpret structured regulatory or biomedical information, supporting their integration into clinical workflows for ICF review.

## Discussion

### Principal Findings

In this study, we have developed a workflow integrating LLMs, augmented with relevant context from ICF documents, to automatically extract and analyze information to support the data reuse process. Our results show that, with expert-defined prompts, our RAG pipeline was able to extract information about data reuse with high accuracy.

To the best of our knowledge, only one study has explored the role of LLMs in analyzing ICF documents for data reuse. This study proposed an adaptable LLM-based framework to aid the analysis of ICFs for data use agreements. They achieved an accuracy of 96.6% with their ICF Q&A chatbot [8]. However, their approach has several limitations: it is designed to process only one ICF at a time, and its evaluation was performed using just 2 ICF documents. As a result, the generalizability and robustness of the chatbot across diverse ICFs, particularly across different languages and countries, remain unclear.

In contrast, we present a comprehensive analysis of more than 900 ICFs to evaluate and validate a novel integrated workflow for ICF review for data reuse. We responsibly leverage a GPT-4.1 LLM augmented with a RAG pipeline to automatically extract information related to data reuse. This workflow provides an intelligent, intuitive, and efficient method for large-scale ICF review. Importantly, our approach supports the simultaneous processing of multiple ICF documents and incorporates a human-in-the-loop design, enabling users to easily review and correct LLM outputs. Our workflow is intentionally human-centered; rather than replacing human reviewers, it aims to enhance the ICF review process by improving scalability and consistency while maintaining expert oversight.

### Findings

We observed a high accuracy rate for most of the data reuse descriptors targeted by our approach. However, poor performance was observed in descriptors such as MED_ALL and RESTRICTIVE_WORDING, highlighting persistent limitations related to ambiguity, edge case reasoning, and underrepresented concepts. The RESTRICTIVE_WORDING descriptor is particularly challenging to identify, even for human reviewers. In contrast to other descriptors, which are clearly defined by words such as “medicine” and “disease,” restrictions on data reuse do not rely on standard phrasings and are therefore intrinsically more difficult to identify. These weaknesses mirror broader concerns regarding LLM hallucinations and misinterpretations in high-stakes settings, reinforcing the need for RAG and other grounding strategies to ensure reliability and safety. Our results also demonstrate that iterative prompt refinement can meaningfully improve performance, as shown in Table 2. Following targeted prompt optimization, average accuracy across data-sharing descriptors increased from 81.6% in the initial evaluation to 92.1% during the final round of validation, indicating that contextual adjustments to prompt structure can yield substantial gains.

In addition, we found that the majority of misclassifications occurred when the expected output value was “N/A” (Figure 4). This pattern suggests that the LLM tends to infer either a confirmation (YES output) or denial (NO output) of data reuse in these cases, even if the document does not contain context to support either decision. Importantly, this behavior did not stem from unsupported hallucinations. For every decision, the model cited specific text from the ICF that influenced its output. During the subsequent review of the referenced passages, subject-matter experts identified recurring phrases in the ICF documents that inadvertently triggered the model’s reasoning. These observations suggest that a small number of linguistic patterns can bypass the intended prompt logic. As such, continuous review of prompt logic vs model output can guide prompt optimization and, thus, reduce misclassification.

Although several individual data-sharing descriptors demonstrated high accuracy, this does not guarantee flawless performance at the document level. In our validation, only 186 of 488 ICFs achieved completely correct outcomes across all descriptors (38.1%), highlighting that even a single incorrect descriptor can lower the overall score. Given that for each ICF 21 descriptors were assessed, the cumulative impact of 1 or 2 errors can substantially reduce the rate of fully correct documents despite strong per-descriptor performance. Overall, our findings suggest that LLMs are well positioned to improve the ICF review process when their outputs are appropriately constrained through well-curated prompts, combined with robust retrieval mechanisms, accounting for nuances in ICF language, and the explicit integration of domain-specific concepts.

### Limitations

Our study is not without limitations. First, after exploring the GPT-4o model, we opted to use GPT-4.1 without exploring other models. We chose GPT-4.1 because it has outperformed other models on several automated experimental tasks [27]. Additionally, GPT-4.1 offers exceptional performance, including instruction following and reasoning, at lower cost [20]. Second, in our study, we used expert-defined prompts to extract relevant information about data reuse. As such, we do not offer chatbot capabilities to allow users to further engage with the document. We chose this approach because we wanted reproducibility in terms of LLM outcomes. Additionally, allowing users to correct the LLM outputs is a more realistic application in high-stakes clinical research where errors are not acceptable, and autonomy remains with the expert user. Third, even with RAG, misinterpretation may still occur, and we found that the LLM still gave wrong information in certain cases because of the inherent nature of how these systems work [28]. Furthermore, as with any automated nondeterministic classification system, false positives and false negatives may occur with our workflow when consent language is ambiguous or context-dependent. As such, we recommend the human-in-the-loop approach to verify LLM outputs regarding data sharing.

However, potential automation bias may arise with the human-in-the-loop workflow, which enables users to review and edit LLM outputs. We did not formally evaluate whether human reviewers consistently detect and correct model errors. Consequently, there is a risk that users may overrely on model-generated outputs and insufficiently scrutinize incorrect or ambiguous results. Mitigation strategies include implementing explicit verification workflows, audits, clear user guidance, and mandatory review steps to ensure that final decisions remain under expert control. Additionally, the absence of an independent, blinded set of external subject-matter experts remains a limitation and may affect the generalizability and robustness of the reported performance.

In our study, we leveraged the multilingual abilities of LLMs, yet we did not formally evaluate translation accuracy. Overreliance on LLMs’ multilingual capabilities may introduce variability in interpretation due to language-specific nuances. Our analyses showed a noticeable difference in the application’s performance from one country to another, which could be due to differences in how the application processes certain languages or writing systems, especially when handling non-Latin scripts or language-specific structures. Despite these limitations, with iterative prompt optimization, our workflow demonstrated the ability to handle ICFs in various languages.

Lastly, during the third round of optimization, we used example phrases within the prompt to guide the LLM to perform complex reasoning [29] and elicit correct classifications. While examples help the model identify semantically similar phrases, using a text fragment drawn from our validation set may limit generalizability. This specific prompt rule could potentially reduce classification performance when evaluating external ICFs with highly unique or divergent wording.

### Future Directions

While our study focuses on analyzing information from ICF documents, in the future we will leverage these insights to standardize ICF creation by accounting for different institutional review boards and country-specific ethical standards to optimize the process end-to-end. Future work will focus on further improvements to our workflow, with minor prompt optimization to capture nuances of language represented across different ICFs to avoid misclassification. Additionally, we will quantify the time and cost-saving impact of automating the ICF review process. While our work illustrates technical feasibility, the deployment of such RAG-based workflows in a regulated clinical setting must ensure compliance with requirements for validation, traceability, and data protection.

### Conclusions

In this study, we have shown that LLMs can be successfully integrated into the clinical workflow for data reuse assessment. Using a zero-shot prompting approach, our results demonstrate that it is possible to automatically extract relevant information about data reuse, such as with whom the data can be shared and for which purpose the data can be reused. This has the potential to increase efficiency, improve accuracy in the ICF review process, and reduce manual effort. In the final round of validation using 104 holdout ICF documents, our novel workflow incorporating GPT-4.1, expert-defined prompts, and hybrid search achieved an accuracy of more than 85% in 19 out of 21 descriptors when compared with human ground truth (Table 2).

Furthermore, our automatic tool helps users understand the nuances of informed consent language by processing ICFs in various languages, including English, German, Dutch, French, Spanish, Korean, and Japanese. This illustrates the scalability of the solution and the adaptability of LLMs to the most common languages. However, challenges such as hallucinations or an LLM using the wrong reasoning may still occur. We have shown that this limitation can be mitigated by incorporating clear examples into prompts. Additionally, using human-in-the-loop oversight allows subject-matter experts to correct errors in the few instances where LLM outputs are inaccurate, thereby ensuring adherence to responsible development and use of AI. In summary, our novel workflow illustrates how LLMs with RAG and zero-shot prompting can be adapted for downstream tasks in clinical research, aiding experts in efficiently reviewing, discerning, and determining appropriate use cases for data reuse.

## Supplementary material

10.2196/95417 Multimedia Appendix 1 The complete list of baseline prompts used in our workflow.

10.2196/95417 Multimedia Appendix 2 Large language model performance comparison between GPT-4o and GPT-4.1.

10.2196/95417 Multimedia Appendix 3 Chunk size comparison.

10.2196/95417 Multimedia Appendix 4 Full outcome from application.

10.2196/95417 Multimedia Appendix 5 Distribution of incorrect large language model outputs during validation.

10.2196/95417 Multimedia Appendix 6 Completely correctly classified informed consent forms (ICFs) from the full validation corpus of 488 ICFs.

## Abbreviations

AWS: Amazon Web Services
BM25: best matching 25
ICF: informed consent form
LLM: large language model
RAG: retrieval-augmented generation
