# Supplementary material for: Large Language Model–Assisted Review of Clinical Trial Informed Consent for Data Reuse: Retrieval-Augmented Generation Approach
Source: JMIR Med Inform. 2026 Oct 15;14:e95417. doi: 10.2196/95417 (PMC13671682; doi:10.2196/95417)
Supplement: Supplementary file 1 [file medinform-v14-e95417-s001.docx]

# **Prompts**

Table S1. The complete list of baseline prompts used in our workflow.

**Prompts**

From the content of the informed consent form (ICF), identify the country associated with the document. The country may be explicitly named or implied through country codes or contextual clues (e.g., regulatory references, language, or location-specific contact details). Spanish language does not necessarily mean Spain is the country associated with the document and requires more thorough assessment. Use the following list of country names and codes as reference: For example: Algeria, AR/ARG (Argentina), AU/AUS (Australia), AT/AUT (Austria), BY (Belarus), BE/BEL (Belgium), Bosnia and Hercegovina, Botswana, BR (Brazil), BG (Bulgaria), CAN (Canada), CL (Chile), CN/CHN (China), CO (Colombia), HR (Croatia), CZ/CZE (Czech Republic), Ecuador, Egyptian (Egypt), EE (Estonia), FI (Finland), FR/FRA (France), GE (Georgia), DE (Germany), GRC (Greece), HU/HUN (Hungary), HKG (Hong Kong), IN/IND (India), ID (Indonesia), IE/IRL/UKIE (Ireland), IL/ISR (Israel), IT/ITA (Italy), JP/JPN (Japan), Jordan, KR/KOR (Korea), LV (Latvia),Lebanon, LT (Lithuania), Macedonia, MY/MYS (Malaysia), MX (Mexico), MD (Moldova), Monaco, Morocco, NLD (Netherlands), NZ/NZL (New Zealand), Norway, Panama, PE (Peru), Philippines, PL/POL (Poland), PT/PRT (Portugal), RO (Romania), RU (Russia), RS (Serbia), SG/SGP (Singapore), SK (Slovakia), SI (Slovenia), South Africa, ES/ESP (Spain), Sri Lanka, SE (Sweden), CH (Switzerland), TW/TWN (Taiwan), Thailand, Tunisia, Turkey, UAE, UA (Ukraine), UK/GBR (United Kingdom), US/USA (United States), Venezuela, Vietnam. Descriptor: COUNTRY

<excerpt from the informed consent form>

{excerpt}

</excerpt from the informed consent form>

From the information provided in the informed consent form (ICF). What is the Trial No (Protocol No) mentioned in the ICF? The Trial No (Protocol No) is in this format: XXXX-XXXX, for example: Trial No: 1443-0004, 1368-0027. Extract where Trial number (Protocol number) is mentioned.

Descriptor: TRIAL_NO

<excerpt from the informed consent form>

{excerpt}

</excerpt from the informed consent form>

From the informed consent form (ICF), extract the version number of the document. Look for phrases like: “version-01”, “version-b-01”, “Main Consent Version # 2.0”, etc. Do not confuse the version number with the version date. Extract the version as a digit only (e.g., 1, 2, 3). Do not include any other characters. Extract information from document to support the answer.

Descriptor: VERSION

<excerpt from the informed consent form>

{excerpt}

</excerpt from the informed consent form>

From the information provided in the informed consent form (ICF). What is the ICF version date? Extract where the date is mentioned. Extract the version date and return in the format DD-MMM-YYYY. For example: 05-Aug-2025. Do not include any other characters.

Descriptor: VERSION_DATE

<excerpt from the informed consent form>

{excerpt}

</excerpt from the informed consent form>

From the informed consent form (ICF), do Licensees (LIC) have access to trial data for reuse — including conducting clinical trials or further developing and commercializing the licensed product under agreed terms? Look for terms like: “transfer,” “transaction,” “merger,” “acquisition,” or references to another organization taking over development. Respond: "YES" if the ICF explicitly allows data access or transfer to licensees or successor entities. "NO" only if it is mentioned that licensees or successor entities explicitly prohibited from accessing or using trial data. "NA" if access is not mentioned or is unclear or no information is available. Extract information from document to support the answer.

Descriptor: LIC

<excerpt from the informed consent form>

{excerpt}

</excerpt from the informed consent form>

From the informed consent form (ICF), do Research Partners or non-commercial Collaborators (RPC) have access to trial data for reuse? Reply "YES" if access is explicitly granted to research partners or collaborators (e.g., “research partners,” “collaborating institutions,” “non-commercial collaborators”). Reply "NO" only if data access by research partners or collaborators (e.g., “research partners,” “collaborating institutions,” “non-commercial collaborators”) is explicitly prohibited. Reply "NA" if access is not mentioned or is unclear or no information is available. Extract information from document to support the answer.

Descriptor: RPC

<excerpt from the informed consent form>

{excerpt}

</excerpt from the informed consent form>

From the informed consent form (ICF), do External Independent Researchers (RPE) have access to trial data for reuse (e.g., to conduct independent studies, validate, challenge, or expand on the sponsor’s findings)? Reply "YES" if the ICF explicitly allows data sharing with independent researchers (e.g., universities, scientific institutions). Reply "NO" only if access by independent researchers (e.g., universities, scientific institutions) is explicitly prohibited. Reply "NA" if access is not mentioned or is unclear or no information is available. Example indicator of "NA" reply: "Please be aware that no data or information can be used for any other research purposes in the future by any means without prior referral to the Research Ethics Committee of the Egyptian Ministry of Health and Population." Extract information from document to support the answer. Descriptor: RPE

<excerpt from the informed consent form>

{excerpt}

</excerpt from the informed consent form>

From the informed consent form (ICF), do Sponsors, affiliates, or third-party processors (SPAFI) have access to trial data for reuse (e.g., analysis, storage, or management)? Reply "YES" if sponsors, affiliates, or third-party processors have access to trial data. Reply "NO" only if access is explicitly prohibited to sponsors, affiliates, or third-party processors to access trial data. Reply "NA" if it is not mentioned that sponsors, affiliates, or third-party processors are granted access to trial data or no information is available. Example indicators of YES reply:

"Your coded data and bio samples are needed for the Sponsor, its research partners and service providers…". "The sponsor, designated personnel, and collaborating organizations may access your data…" Example indicator for NA reply:

"The samples or parts of them may be transferred to the sponsor, its research partners and service providers (like clinical research organizations or laboratories) including companies belonging to the Boehringer Ingelheim Group of Companies." Extract information from document to support the answer.

Descriptor: SPAFI

<excerpt from the informed consent form>

{excerpt}

</excerpt from the informed consent form>

From the information provided in the informed consent form (ICF). Can trial data be used to improve medication quality and related substances? This includes understanding the medication's safety, efficacy, pharmacokinetics, pharmacodynamics, and interactions. Look for phrases such as: "understand how the trial drug work in the body and the study drug mode of action" If it is mentioned that data can be reused to understand how trial drug works, then reply YES. If it is explicitly mentioned that data "cannot" be used to understand how trial drug works, reply NO. If it is not explicitly mentioned that data can be used to understand how the trial drug works or no information is available, reply NA. Extract information from document to support the answer.

Descriptor: MED_TR

<excerpt from the informed consent form>

{excerpt}

</excerpt from the informed consent form>

From the information provided in the informed consent form (ICF).

Can trial data be used to improve medication quality and related substances? This info is in the confidentiality and data privacy section.

Look for phrases such as: "understand how the trial drug and similar drugs work in the body and the study drug mode of action"

If it is mentioned that data can be reused to understand how similar trial drug works or better understand related diseases, then reply YES.

If it is explicitly mentioned that data "cannot" be used to improve related medication or similar trial drugs, reply NO.

If it is not explicitly mentioned that data can be used to understand similar trial drugs or related medication or no information is available, reply NA.

Extract information from document to support the answer. Descriptor: MED_TR_REL

<excerpt from the informed consent form>

{excerpt}

</excerpt from the informed consent form>

From the informed consent form (ICF), can trial data be used to improve the quality or understanding of any medications or substances? This includes safety, efficacy, pharmacokinetics, pharmacodynamics, and interactions. Reply: "YES" if the ICF clearly allows data use for understanding or improving any medications or substances.

"NO" only if it explicitly stated that use of data to improve or understand any medication or any trial drugs is not allowed.

"NA" if this is not mentioned or is unclear or no information is available. Example indicator of "NA" reply: "Please be aware that no data or information can be used for any other research purposes in the future by any means without prior referral to the Research Ethics Committee of the Egyptian Ministry of Health and Population." Extract information from document to support the answer.

Descriptor: MED_ALL

<excerpt from the informed consent form>

{excerpt}

</excerpt from the informed consent form>

From the informed consent form (ICF), can trial data be reused broadly to study the trial disease and any other diseases (not just related ones)? Reply "YES" if the ICF explicitly allows reuse to understand other diseases, associated illnesses, risk factors, or affected populations (e.g., “to better understand yours and other diseases”). Reply "NO" only if the broad use of data to understand other diseases is explicitly prohibited. Reply "NA" if it is not mentioned that data can be used to understand any other diseases or no information is available. Extract information from document to support the answer. Descriptor: DIS_ALL

<excerpt from the informed consent form>

{excerpt}

</excerpt from the informed consent form>

From the information provided in the informed consent form (ICF). Can trial data be reused to learn about the trial disease? This includes detailed analysis of the disease's nature, progression, and impact on the trial's patient population. Example: "better understand yours, related diseases and associated health problems" If it is mentioned that data can be use to allow for detailed analysis to provide more understanding of the disease's nature, progression, and impact on the trial's patient population reply YES. If it is explicitly mentioned that data "cannot" be used to understand the disease's nature, progression and impact on trial's patient population, reply NO. If it is not explicitly mentioned that data can be used to understand the disease's nature, progression, and impact on the trial's patient population or no information is available, reply NA. Extract information from document to support the answer.

Descriptor: DIS_TR

<excerpt from the informed consent form>

{excerpt}

</excerpt from the informed consent form>

Based on the informed consent form (ICF), can trial data be reused to understand the trial disease and related conditions? Reply "YES" if the ICF explicitly allows data use to study the trial disease and related or similar diseases (e.g., "to better understand your disease and related health problems"). Reply "NO" if it explicitly prohibits such use. Reply "NA" if this is not mentioned or is unclear. Extract information from document to support the answer.

Descriptor: DIS_TR_REL

<excerpt from the informed consent form>

{excerpt}

</excerpt from the informed consent form>

From the informed consent form (ICF), can trial data be reused to study the trial disease or other diseases within specific therapeutic areas (e.g., cardiovascular, oncology, immunotherapy, respiratory)? Respond based on the following criteria:

Reply "YES" if the ICF explicitly allows reuse to understand diseases within these or other defined therapeutic areas. Reply "NO" only if it is mentioned that data "cannot" be used to understand other diseases within the defined therapeutic area/areas. Reply "NA" if the use of data to understand diseases within these or other defined therapeutic areas is not mentioned or is unclear. Extract information from document to support the answer.

Descriptor: DIS_TR_TA

<excerpt from the informed consent form>

{excerpt}

</excerpt from the informed consent form>

From the informed consent form (ICF), determine whether trial data can be broadly reused to support the development of any diagnostic products — including unrelated ones — such as new technologies, improved tools, or diagnostic strategies.

Respond based on the following criteria:

Reply "YES" if the ICF explicitly allows data reuse for any diagnostic product development.

Reply "NO" if only the ICF explicitly prohibits data reuse specifically for any diagnostic product development. Reply "NA" if the ICF does not mention use of data for any diagnostic product development or is unclear or no information available. Example indicator of NA reply: "Please be aware that no data or information can be used for any other research purposes in the future by any means without prior referral to the Research Ethics Committee of the Egyptian Ministry of Health and Population." Extract information from document to support the answer.

Descriptor: DPROD_ALL

<excerpt from the informed consent form>

{excerpt}

</excerpt from the informed consent form>

From the information provided in the informed consent form (ICF). Can trial data be reused to develop diagnostic products related to the trial disease? This includes creating, testing, and refining diagnostic tools, devices, or procedures specifically for the disease under investigation.

Example: "develop diagnostic tests for, or drugs to treat yours and related diseases."

If it is mentioned that data can be reused to support the development of diagnostic products related to trial disease, reply YES.

If it is explicitly mentioned that data "cannot" be used to support the development of "related" or "similar" diagnostic products is explicitly forbidden, reply NO.

If it is not explicitly mentioned that data can be used to support the development of "related" or "similar" diagnostic products or no information is available, reply NA.

Examples indicators of NA reply: "Please be aware that no data or information can be used for any other research purposes in the trial". "Please be aware that no data or information can be used for any other research purposes in the future by any means without prior referral to the Research Ethics Committee of the Egyptian Ministry of Health and Population." Extract information from document to support the answer. Descriptor: DPROD_TR_REL

<excerpt from the informed consent form>

{excerpt}

</excerpt from the informed consent form>

From the information provided in the informed consent form (ICF). Does the ICF mention that trial data can be reused to develop other types of therapeutic products, tools, devices, or procedures, including new treatments (not only related trial)?

If it is mentioned that data can be reused to support the development of any other types of therapeutic products, reply YES.

If it is explicitly mentioned that data "cannot" be used to support the development of any other types of therapeutic products, tools, devices or procedures, including new treatments is explicitly forbidden, reply NO.

If it is not explicitly mentioned that data can be used to support the development of any other types of therapeutic products, tools, devices or procedures, including new treatments or no information is available, reply NA.

Examples indicators of NA reply:

"Please be aware that no data or information can be used for any other research purposes in the procedures which are not known at this time."

"Please be aware that no data or information can be used for any other research purposes in the future by any means without prior referral to the Research Ethics Committee of the Egyptian Ministry of Health and Population."

Extract information from document to support the answer.

Descriptor: TRPROD_ALL

<excerpt from the informed consent form>

{excerpt}

</excerpt from the informed consent form>

From the information provided in the informed consent form (ICF). Does the ICF allow data reuse to develop therapeutic products related to the trial disease?

If it is mentioned that data can be reused to support the development of therapeutic tools and procedures related to the disease being studied in a clinical trial, reply YES. If it is explicitly mentioned that data "cannot" be used to support the development of therapeutic tools and procedures related to the disease being studied in a clinical trial is explicitly forbidden, reply NO. If it is not explicitly mentioned that data can be used to support the development of related of therapeutic products, tools, devices or procedures, including new treatments or no information is available, reply NA. Example indicator of "NA" reply: "Please be aware that no data or information can be used for any other research purposes in the future by any means without prior referral to the Research Ethics Committee of the Egyptian Ministry of Health and Population." Extract information from document to support the answer.

Descriptor: TRPROD_TR_REL

<excerpt from the informed consent form>

{excerpt}

</excerpt from the informed consent form>

From the information provided in the informed consent form (ICF). Does the ICF mention that trial data can be used to improve the quality of this and other trials? This includes learning from past studies to inform future trial design and enhance scientific analysis methods. Look for the words "to improve quality".

For example, "learn from past studies to plan new studies or improve scientific analysis methods". If it is mentioned that data can be reused to improve quality of this and other trails, reply YES. If it is explicitly mentioned that data "cannot" be used to improve quality of this and other trails reply NO. If it is not explicitly mentioned that data can be used to improve quality of this and/or other trails or if no information is available reply with NA. Extract information from document to support the answer.

Descriptor: QUAL_ALL

<excerpt from the informed consent form>

{excerpt}

</excerpt from the informed consent form>

From the information provided in the informed consent form (ICF).

Does the ICF mention if the link for re-identification of data subject will be deleted in 30 years? Check for word 'link', implying link between data and IDs.

Examples: "All coded data, including yours, will be kept by the Sponsor. Only your trial doctor will be able to link your unique code number to you. This link will remain at the trial site for a maximum of 30 years and will then be destroyed by the trial doctor. After that it is not possible to link your unique code number directly back to you." - "This link will remain at the trial site for a maximum of 30 years and will then be destroyed by the trial doctor." "While the data can be collated in medical institutions for a maximum of 30 years after the completion of the clinical trial, the data that the investigator can collate will be discarded after that period. After that, you will not be able to directly match your unique code number."

The answer can be only YES or NO. If it is mentioned that the link for re-identification of data subject will be deleted in 30 years, reply YES, else reply NO.

Extract information from document to support the answer. Descriptor: DLINK30

<excerpt from the informed consent form>

{excerpt}

</excerpt from the informed consent form>

From the information provided in the informed consent form (ICF). Does the ICF mention if data can be used for up to 50 years? Answer can be only YES or NO. If the data can be stored for 50 years, respond with YES. Else respond with NO. Extract information from document to support the answer.

Descriptor: RR50

<excerpt from the informed consent form>

{excerpt}

</excerpt from the informed consent form>

From the information provided in the informed consent form (ICF). Does the ICF mention if data can be used for up to 80 years? Answer can be only YES or NO. If the data can be stored for 80 years, respond with YES. Else respond with NO. Extract information from document to support the answer.

Descriptor: RR80

<excerpt from the informed consent form>

{excerpt}

</excerpt from the informed consent form>

From the information provided in the informed consent form (ICF). Does the ICF contain sentences with the same implied meaning as any of the following examples? Examples:

- "Furthermore, law number 19.628 will be followed strictly."

- "All personal information regarding your participation in this study will be confidential, with the exception of cases in which access is required by law."

- "Additional research outside of the trial using encoded data must be approved by the ethics committee."

- "Prior approval by the ethics committee is required if an external researcher uses data for a project outside the scope of the ICF."

- "Additional research outside of the trial using encoded data must be approved by the ethics committee." The answer can be only YES or NO. If any of the above examples or similar appear, respond with YES. Else if not reply with NO.

Descriptor: RESTRICTIVE_WORDING

<excerpt from the informed consent form>

{excerpt}

</excerpt from the informed consent form>

From the information provided in the informed consent form (ICF). Does the ICF mention Genomic information. Look for mentions of genes, genetic testing, DNA, or RNA. Examples of phrases that imply genomic information:

1. For ICFs in Dutch ICFs, look for the following examples:

- “Uit het lichaamsmateriaal dat we voor dit extra onderzoek bij u afnemen halen we de erfelijke informatie”. “Deze stofjes kunnen bijvoorbeeld eiwitten, RNA of DNA zijn. RNA en DNA bevat erfelijke informatie.”

2. English specific examples:

- Your DNA (genetic information) will be removed from one blood sample

- In this trial, non-genetic and genetic biomarker testing will be done

- DNA genetic tests will be conducted to evaluate specific genes known to cause mutations changes in the gene structure) that may lead to GPP. RNA tests will be conducted to identify genes involved in how the investigational drug works in the body, how the body responds to the drug, and the severity of the disease. “DNA (genetic information) will be removed 25 years data retention period. I understand that genetic analysis will be conducted on my biological samples.”

- "If genetic studies are done on the samples you provide, they may involve looking at just a part of your genetic information. Or, these studies may involve reading the entire genetic instruction book, or all of your genetic information (called whole genome sequencing)."

- "Your clinical data will be stored as described in the information and consent form for the main clinical trial."

- "Individual research data obtained by the analysis of your samples and/or your related clinical data will be stored for up to thirty (30) years after they have been obtained."

- Other non-genetic biomarker tests may also be conducted, but it is not yet known what tests will be performed.

- Genetic DNA tests will be conducted to evaluate specific genes known to have mutations (changes in the structure of the gene) that may cause PPG.

- PG testing will look at parts of DNA (deoxyribonucleic acid), at RNA (ribonucleic acid), or at other gene products, like proteins in your cells

- Genetic tests will be performed to identify genes from RNA that are involved in how the investigational drug works in the body, how the body responds to the drug, and the severity of the disease.

3. Other languages, identify phrases that may imply genomic information, DNA/RNA etc. If words such as: genes, genetic testing, genetic, genetic information, DNA, or RNA or similar are mentioned, reply YES. Else if no mention of words such as: genes, genetic testing, genetic, genetic information, DNA, or RNA, reply NO.

Descriptor: GENOMIC_INFO

<excerpt from the informed consent form>

{excerpt}

</excerpt from the informed consent form>
