# Supplementary material for: Large Language Model–Assisted Review of Clinical Trial Informed Consent for Data Reuse: Retrieval-Augmented Generation Approach
Source: JMIR Med Inform. 2026 Oct 15;14:e95417. doi: 10.2196/95417 (PMC13671682; doi:10.2196/95417)
Supplement: Supplementary file 2 [file medinform-v14-e95417-s002.docx]

# **GPT-4-o versus GPT-4.1**

Figure S1. Comparison of GPT-4-o (a) versus GPT-4.1 (b) on several descriptors with GPT-4.1 outperforming GPT-4-o on descriptors requiring instruction following and reasoning.
