# Supplementary material for: Large Language Model–Assisted Review of Clinical Trial Informed Consent for Data Reuse: Retrieval-Augmented Generation Approach
Source: JMIR Med Inform. 2026 Oct 15;14:e95417. doi: 10.2196/95417 (PMC13671682; doi:10.2196/95417)
Supplement: Supplementary file 3 [file medinform-v14-e95417-s003.docx]

# **Chunk size comparisons**

Table S2. Performance of the different chunk sizes terms of accuracy (%) for all data sharing descriptors. The performance was conducted on 438 ICFs.

| **Descriptor** | **500** | **1000** | **2000** |
| --- | --- | --- | --- |
| **LIC** | 78.54 | 86.99 | 91.1 |
| **RPC** | 70.55 | 72.83 | 78.31 |
| **RPE** | 76.94 | 82.42 | 88.81 |
| **SPAFI** | 85.62 | 86.3 | 88.13 |
| **MED_TR** | 87.67 | 87.67 | 89.04 |
| **MED_TR_REL** | 85.62 | 88.36 | 89.04 |
| **MED_ALL** | 63.7 | 58.45 | 57.99 |
| **DIS_ALL** | 88.81 | 85.16 | 81.74 |
| **DIS_TR** | 85.39 | 88.36 | 88.81 |
| **DIS_TR_REL** | 84.47 | 87.44 | 88.36 |
| **DIS_TR_TA** | 63.47 | 58.45 | 57.99 |
| **DPROD_ALL** | 82.19 | 80.14 | 73.29 |
| **DPROD_TR_REL** | 85.16 | 89.5 | 93.15 |
| **TRPROD_ALL** | 80.59 | 76.71 | 71.46 |
| **TRPROD_TR_REL** | 83.11 | 86.76 | 91.32 |
| **QUAL_ALL** | 84.25 | 85.62 | 90.64 |
| **DLINK30** | 77.63 | 77.17 | 77.85 |
| **RR50** | 91.32 | 92.47 | 92.69 |
| **RR80** | 94.29 | 94.52 | 94.52 |
| **RESTRICTIVE_WORDING** | 59.13 | 61.87 | 64.61 |
| **GENOMIC_INFO** | 62.33 | 63.7 | 65.07 |
| **Overall** | **79.6** | **80.5** | **81.6** |
