# Supplementary material for: Large Language Model–Assisted Review of Clinical Trial Informed Consent for Data Reuse: Retrieval-Augmented Generation Approach
Source: JMIR Med Inform. 2026 Oct 15;14:e95417. doi: 10.2196/95417 (PMC13671682; doi:10.2196/95417)
Supplement: Supplementary file 4 [file medinform-v14-e95417-s004.docx]

# **Full outcome from the Application**

A screenshot of the complete outcome of the application.

Figure S2. The full outcome from the applications.
