# Supplementary material for: Large Language Model–Assisted Review of Clinical Trial Informed Consent for Data Reuse: Retrieval-Augmented Generation Approach
Source: JMIR Med Inform. 2026 Oct 15;14:e95417. doi: 10.2196/95417 (PMC13671682; doi:10.2196/95417)
Supplement: Supplementary file 5 [file medinform-v14-e95417-s005.docx]

# **Incorrect LLM output breakdown**

Figure S3. Distribution of LLM incorrect outputs during validation rounds: a) Baseline – High initial error rates across multiple descriptors, especially MED_ALL and RESTRICTIVE_WORDING. b) First round – early improvements. However, only 14 ICFs tested. c) Second iteration – larger validation of more than 200 ICFs. d) After final optimisation round, validation on 104 ICFs showing overall stabilisation with lower errors, though some descriptors still show residual issues.
