# Supplementary material for: Large Language Model–Assisted Review of Clinical Trial Informed Consent for Data Reuse: Retrieval-Augmented Generation Approach
Source: JMIR Med Inform. 2026 Oct 15;14:e95417. doi: 10.2196/95417 (PMC13671682; doi:10.2196/95417)
Supplement: Supplementary file 6 [file medinform-v14-e95417-s006.docx]

# **Completely correct ICFs**

Table S3. The number of the full validation corpus of 488 ICFs analysed grouped by country, including the number of ICFs which were completely correct and the number of ICFs where the extracted outcome on data sharing descriptors were correct. OPUs= operative units (templates).

| **Country** | **Total** | **ICFs Data Sharing Correct** | **%** |
| --- | --- | --- | --- |
| OPUs | 47 | 13 | 27.7 |
| Argentina | 3 | 2 | 66.7 |
| Austria | 35 | 13 | 37.1 |
| Belgium | 4 | 2 | 50.0 |
| Canada | 14 | 2 | 14.3 |
| Chile | 4 | 0 | 0.0 |
| China | 12 | 2 | 16.7 |
| Czech Republic | 14 | 1 | 7.1 |
| Denmark | 23 | 3 | 13.0 |
| Germany | 44 | 24 | 54.5 |
| Hungary | 6 | 4 | 66.7 |
| Ireland | 9 | 7 | 77.8 |
| Italy | 5 | 0 | 0.0 |
| Japan | 15 | 1 | 6.7 |
| Republic of Korea | 51 | 6 | 11.8 |
| Mexico | 7 | 2 | 28.6 |
| Netherlands | 20 | 5 | 25.0 |
| Norway | 10 | 10 | 100.0 |
| Poland | 39 | 32 | 82.1 |
| Portugal | 4 | 1 | 25.0 |
| Russia | 12 | 7 | 58.3 |
| Spain | 31 | 12 | 38.7 |
| Sweden | 2 | 0 | 0.0 |
| Taiwan | 13 | 8 | 61.5 |
| Turkey | 2 | 2 | 100.0 |
| United Kingdom | 32 | 9 | 28.1 |
| USA | 30 | 18 | 60.0 |
|  |  |  |  |
| **TOTAL** | **488** | **186** |  |
